# Relationship Between Number of Oocytes Retrieved and Embryo Euploidy Rate in Controlled Ovarian Stimulation Cycles

**DOI:** 10.1007/s43032-022-01017-7

**Published:** 2022-08-23

**Authors:** Jonathan D. Buerger, Jitesh Datla, Shahab Minassian, Sarah Dreibelbis, Michael J. Glassner, John J. Orris, Nicolle Clements, Alyssa Sheffy, Sharon H. Anderson

**Affiliations:** 1 Tower Health Reading Hospital, West Reading, PA USA; 2 Saint Joseph’s University, Philadelphia, PA USA; 3 Main Line Fertility Center, Bryn Mawr, PA USA

**Keywords:** Euploid embryos, Retrieved oocytes, Controlled ovarian stimulation, In vitro fertilization, Preimplantation genetic testing for aneuploidy

## Abstract

This cohort study is aimed to determine if higher number of oocytes retrieved affects the rate of euploidy in the embryos of women undergoing controlled ovarian stimulation (COS) for in vitro fertilization (IVF) with preimplantation genetic testing for aneuploidy (PGT-A). A negative trend between the number of oocytes retrieved and embryo euploidy rate was observed using Visual Analytics software, especially when a higher number of oocytes were retrieved. After regression analysis, patient age was the only variable found to have a statistically significant negative effect (*p* < 0.0001) on euploidy rate in all regression models. Number of oocytes retrieved was not found to have a statistically significant effect on euploidy rate when analyzed per number of biopsied blastocysts (*p* = 0.5356), per number of oocytes retrieved (*p* = 0.1025), and per number of fertilized oocytes (*p* = 0.7241). The parameter estimates in the linear regression models were negative for number of oocytes retrieved. This study shows a statistically significant effect between patient age and embryo euploidy rate, which is already known. There is some evidence to suggest that higher number of oocytes retrieved may negatively impact the number of euploid embryos per number of oocytes retrieved based on the visual analytic graphs, *p* value approaching significance, and the negative parameter estimates in the regression models.

## Introduction

A major if not ultimate objective of artificial reproductive technologies (ART) is to maximize the live birth rate. Euploid embryos have been postulated to provide an optimal chance of achieving a live birth, particularly in women of advanced maternal age whose likelihood of aneuploidy is higher than that of their younger counterparts. Thus, attaining a maximum number of euploid embryos projects as a desirable goal. Following this logic, retrieving a larger number of oocytes theoretically should lead to a higher number of euploid embryos after fertilization. This generalization drives controlled ovarian stimulation (COS) protocols to maximize the number of retrieved oocytes while still balancing the associated risks of COS such as ovarian hyperstimulation syndrome (OHSS) with estimated incidences of moderate and severe OHSS at 3–6% and 0.1–2%, respectively, in patients undergoing in vitro fertilization (IVF) [1].

The question arises as to the relationship between the quantity of retrieved oocytes and subsequent quality of fertilized embryos. Studies in animal models have raised concern regarding ovulation induction and subsequent embryo quality. An animal model in mice showed a delay in embryonic development as well as increased abnormal blastocyst formation after ovarian stimulation [2]. Increase in aneuploidy rates during COS may be attributed to enhancing abnormal segregation of chromosomes during meiosis through accelerating nuclear maturation resulting in abnormal chromosomes [3]. Animal model findings, however, have not been consistently seen in clinical human studies. Some clinical studies have analyzed certain factors and their effect on embryo euploidy. Female age has been shown to have an inverse relationship on embryo euploidy [4]. Follicle size has been shown to be a valuable predictor of oocyte maturity and ability of fertilized oocytes to become quality blastocysts; however, follicle size itself has not been shown to reliably predict the euploidy of each quality blastocyst [5]. Anti-Mullerin hormone (AMH) is a hormone produced by the granulosa cell of preantral and small antral follicles. Often used as a screening test for diminished ovarian reserve with lower values having good specificity for poor response to ovarian stimulation, positive relationships between AMH level and the rate of euploid blastocysts have been observed [6]. However, this can be confounded by certain conditions, such as polycystic ovarian syndrome (PCOS) and obesity, where higher AMH levels may actually be indicative of lesser quality oocytes [7].

There is previous evidence to suggest that number of oocytes retrieved does not influence embryo ploidy rate or live birth rates when stratified per the woman’s age at time of egg retrieval [3]. Further exploration of the relationship between increased number of oocytes at time of retrieval and subsequent embryo development including euploidy can have significant impact on future COS protocols. The objective of this study is to determine if higher number of oocytes retrieved affects the rate of euploidy in subsequent embryos.

## Materials and Methods

The Main Line Fertility Center located in Bryn Mawr, Pennsylvania, is a private fertility practice that provides both clinical and laboratory services for patients undergoing assisted reproductive technology (ART). Over the years, a large cohort of reproductive cycles, including oocyte retrieval with subsequent fertilization and preimplantation genetic testing of embryos, has been completed. From these cycles, pertinent data points have been collected to form an extensive data base. A cohort study was completed using data from 902 patients who underwent COS for IVF with PGT-A from 2017 to 2019.

Subsequent exploratory analysis focused mainly on the association between oocyte retrieval number and embryo euploidy rates. When assessing embryo euploid rates, only grade A and B blastocytes were biopsied and tested for euploidy with euploid rate determined by number of euploid embryos out of the total number of grade A and B blastocytes biopsied. Interactive real-time graphing software, Tableau, was used to plot the number of oocytes retrieved (independent variable) and embryo euploidy rate (dependent variable). Several additional models were developed to analyze the effect of dependent variables (age, AMH level, and genetic testing lab) on the outcome variable (euploidy rate) using one-way ANOVA analysis and *t* test.

Multiple linear regression models were developed to evaluate the relationship between number of oocytes retrieved and euploidy rate, using different variations of the outcome (euploidy rate) and dependent variables (number of biopsied blastocysts, number of oocytes retrieved, number of fertilized oocytes) while keeping the independent variables (patient age, AMH level, genetics lab that did the testing, number of oocytes retrieved) constant. Statistical significance was determined by *p* < 0.05.

## Results

**Fig. 1:**
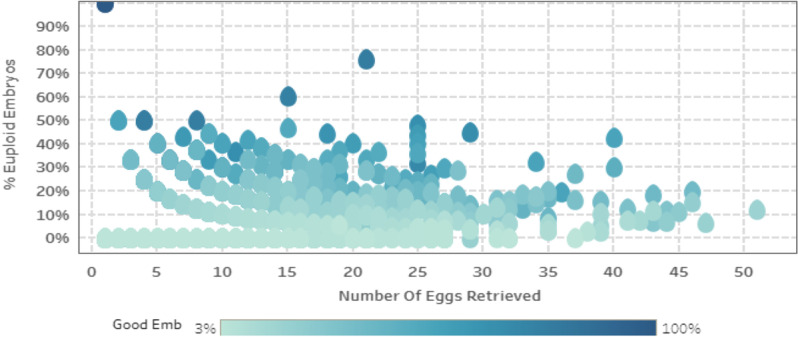
Interactive Tableau software used to graph number of oocytes retrieved versus percent of euploid embryos with darker shades of blue indicating higher percentage of good quality embryos

The number of oocytes retrieved ranged from 1 to 63. An overall negative trend was observed between the number of oocytes retrieved and embryo euploidy rate when the interactive Tableau graphing software was used for visual analytics (Fig. 1). Furthermore, color coordination was used within the Tableau graphing software with darker color shades indicating a greater percentage of “good” embryos obtained with “good” embryos defined as grade A and B blastocytes. A higher-valued percentage of good embryos, or darker color–shaded data points, were more concentrated on the graph where the number of eggs retrieved was low (Fig. 1).

**Fig. 2:**
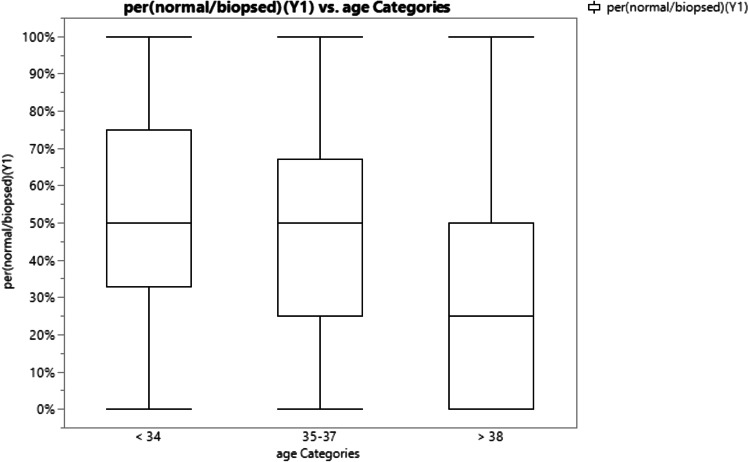
Visual representation of mean percentages of euploid embryos per number of biopsies blastocytes in 3 age group categories

**Fig. 3:**
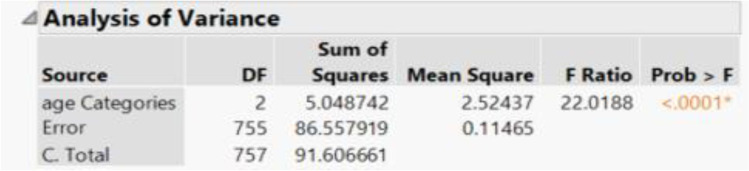
One-way ANOVA analysis results comparing mean percentages of euploid embryos per number of biopsies blastocysts in 3 age group categories

When comparing the effect of patient age on percentage of euploid embryos, age groups were stratified into patient age less than 34 years, 35–37 years, and greater than 38 years. The average percent euploid rates of embryos for the three age groups, respectively, were 53.5%, 47.4%, and 34.3% (Fig. 2). One-way ANOVA analysis revealed a statistically significant difference (*p* value < 0.0001) between age and euploid rate (Fig. 3).

**Fig. 4:**
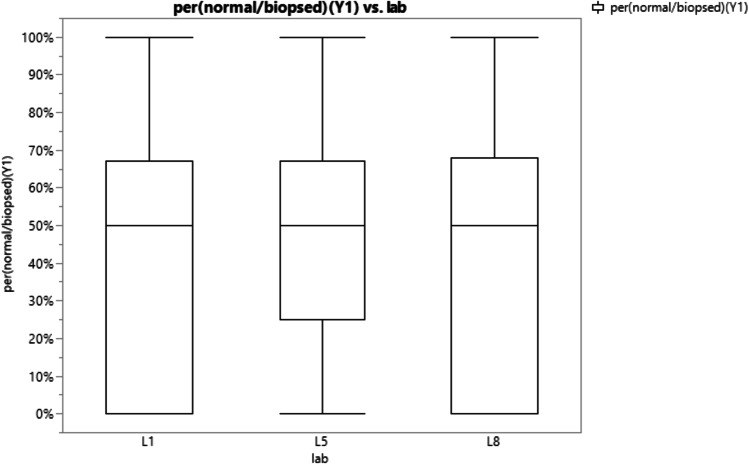
Visual representation of mean percentages of euploid embryos per number of biopsies blastocytes at 3 different PGT testing lab sites

**Fig. 5:**
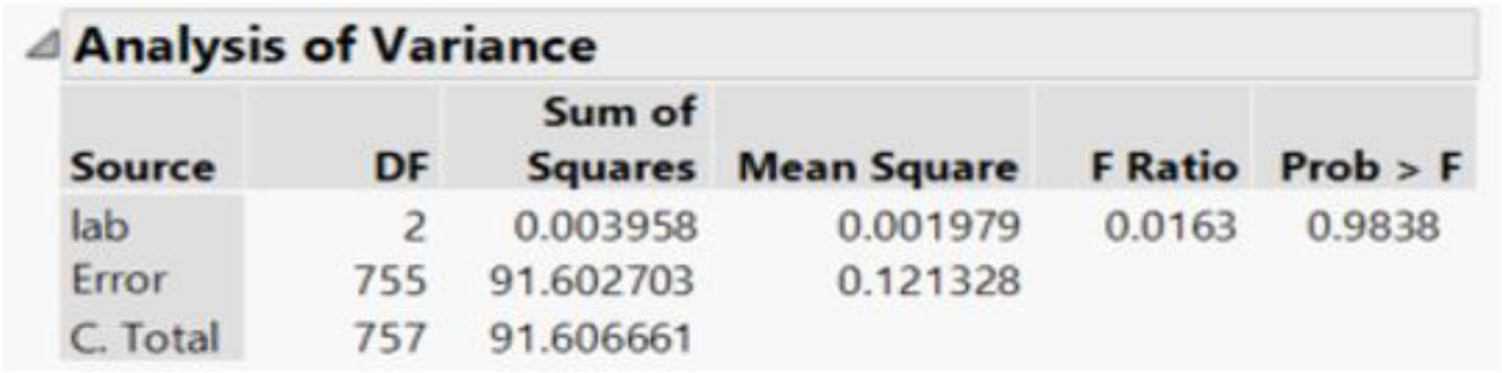
One-way ANOVA analysis results comparing mean percentages of euploid embryos per number of biopsies blastocysts at 3 PGT testing labs

Three labs with the ability to perform PGT-A were used for comparison, where the percentage of euploid embryos was calculated based on the number of biopsied blastocysts. The average percentage of euploid embryos at the respective genetic testing labs were 44.3%, 44.5%, and 44.8% (Fig. 4). One-way ANOVA did not show significant difference (*p* value 0.9838) between the compared labs and euploid rate (Fig. 5).

**Fig. 6:**
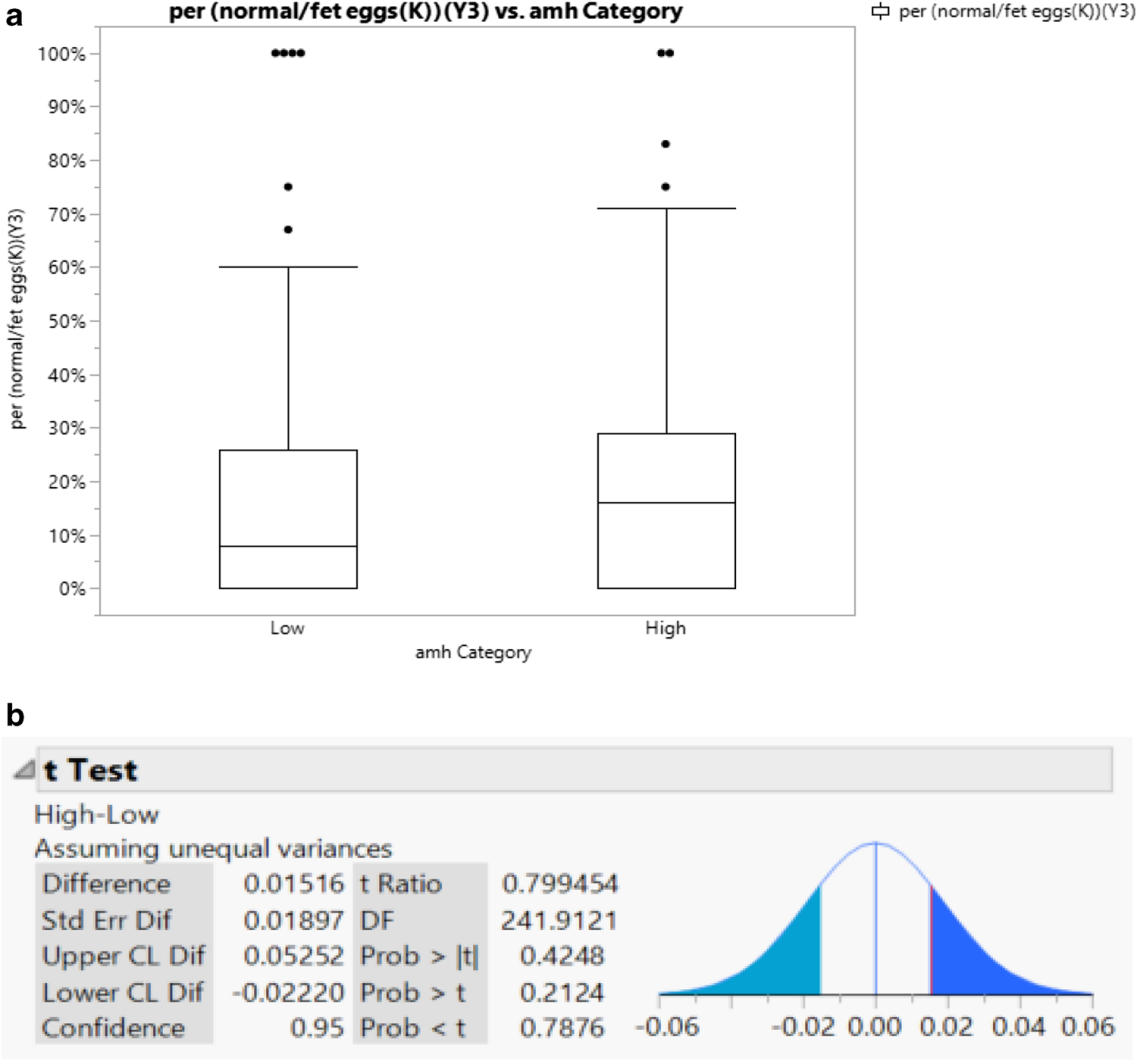
**a** Visual representation of mean percentages of euploid embryos per number of biopsied blastocysts in low AMH group (less than 1) and high AMH group (greater than and equal to 1). **b** *T* test comparing mean percentages of euploid embryos per number of biopsies blastocysts in low AMH group (less than 1) and high AMH group (greater than and equal to 1)

AMH level was dichotomized into a binary variable with “low” AMH classified as a value less than 1 ng/ml and “high” AMH as a value greater than or equal to 1 ng/ml. A *t* test was performed between the two groups which did not show a statistically significant difference (*p* value 0.4248) in the percent of euploid embryos between the low and high AMH groups (Fig. 6).

**Fig. 7:**
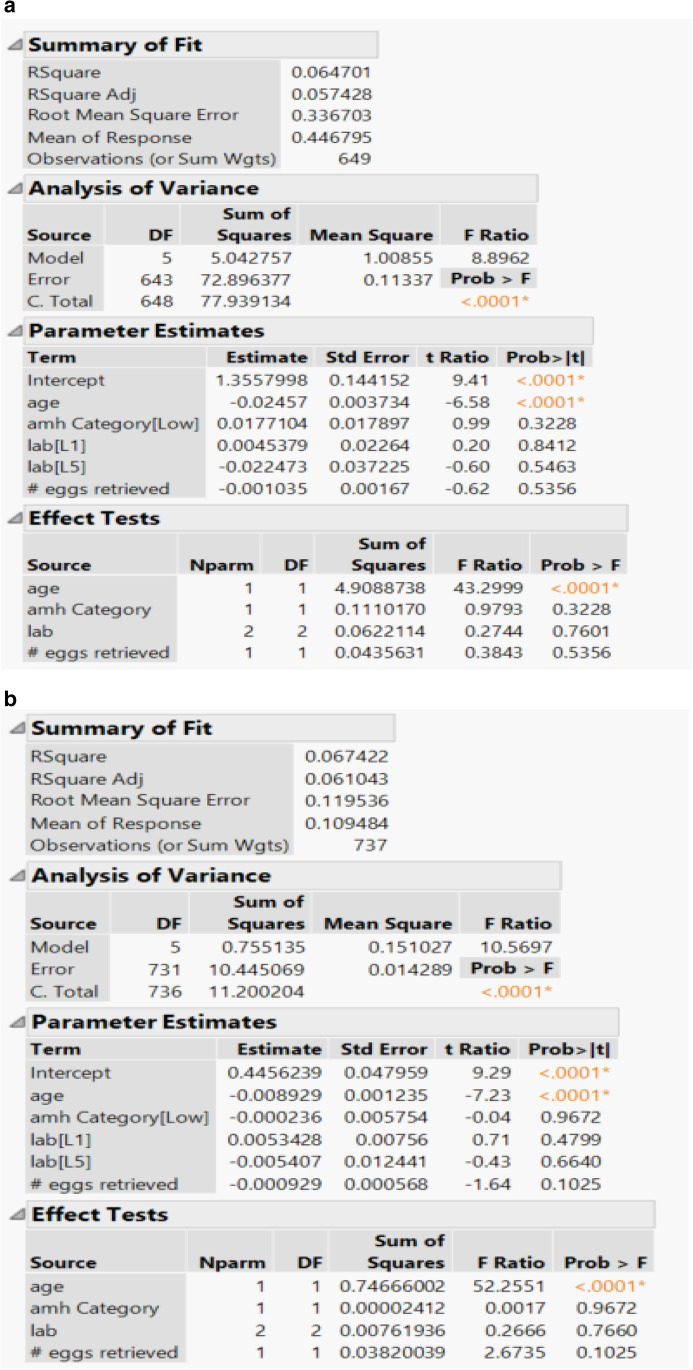

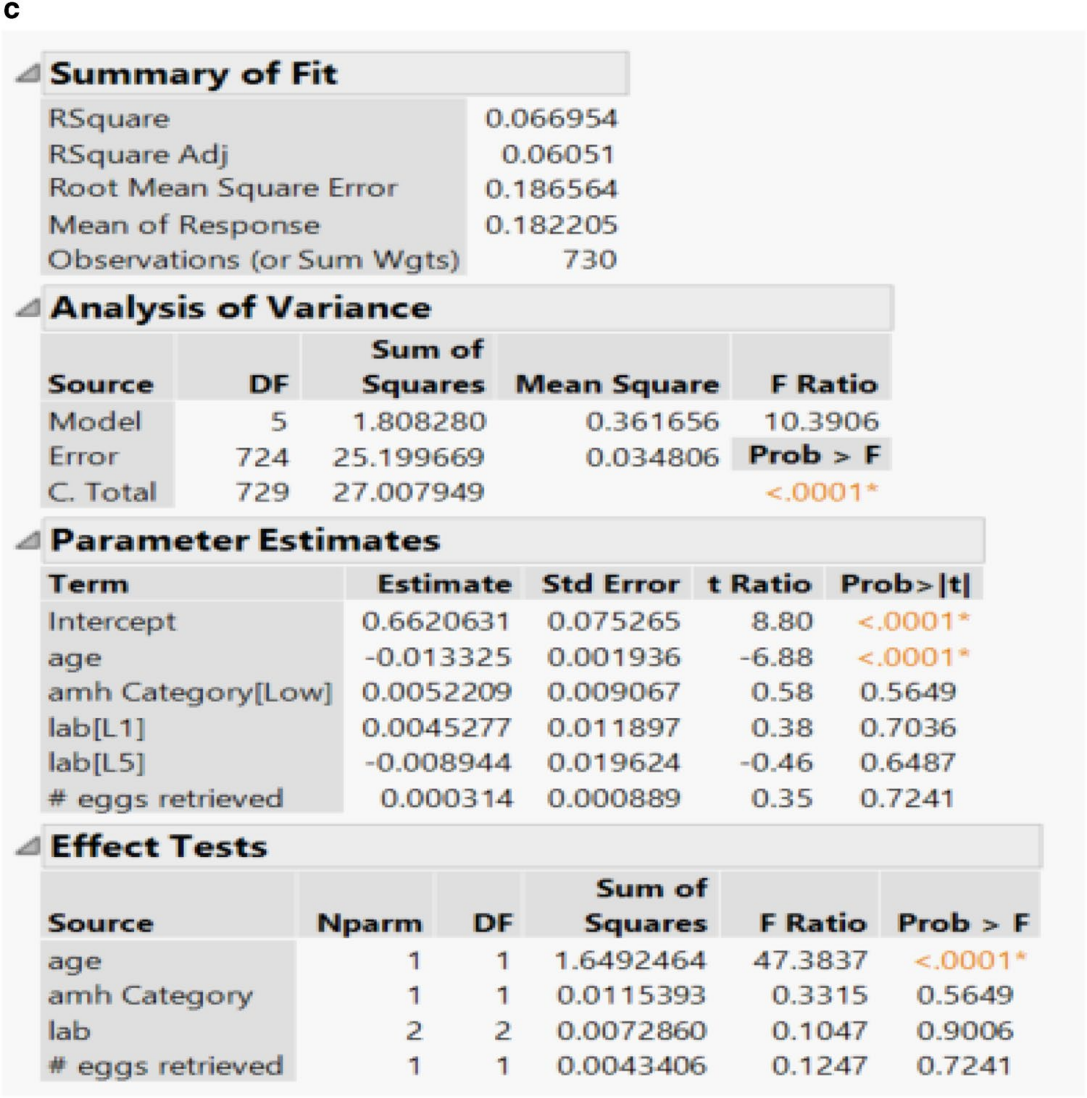
**a** Linear regression analysis using number of euploid embryos as outcome variable and number of biopsied blastocytes as dependent variable with independent variables of patient age, AMH level, PGT testing lab, and number of oocytes retrieved. **b** Linear regression analysis using number of euploid embryos as outcome variable and number of oocytes retrieved as dependent variable with independent variables of patient age, AMH level, PGT-A testing lab, and number of oocytes retrieved. **c** Linear regression analysis using number of euploid embryos as outcome variable and number of fertilized oocytes as dependent variable with independent variables of patient age, AMH level, PGT-A testing lab, and number of oocytes retrieved

After linear regression analysis, patient age was found to be a significant variable (*p* value < 0.0001) in relation to euploidy rate in all three regression models (Fig. 7). Two PGT testing labs were included in the linear regression analysis with neither proving to be statistically significant compared to euploidy rate in any of the regression models. AMH level (*p* value 0.3228, 0.9672, 0.5649) and number of oocytes retrieved (*p* value 0.5356, 0.1025, 0.7241) were not found to be significant variables in relation to euploidy rate in any of the three regression models, respectively. Two of the three regression models did show negative parameter estimates (− 0.001035, − 0.000929) when looking at the number of oocytes retrieved.

## Discussion

A statistically significant effect was not found between the number of oocytes retrieved and euploidy rates of patients who underwent COS for IVF cycles using PGT-A. Furthermore, there was not a statistically significant effect seen between the additional variables of AMH level or PGT testing labs on euploidy rate in the regression analysis. There was a statistically significant effect observed in the regression analyses between patient age and embryo euploidy rate, which is already known.

Although a statistically significant effect was not seen when comparing oocyte retrieval number to embryo euploidy rate, there was some evidence to suggest that higher number of oocytes retrieved may negatively impact the rate of embryo euploidy. An intriguing negative trend in euploidy rate was observed in the Tableau Visual Analytics graphs, especially when the number of oocytes retrieved was noted to be on the higher end of the spectrum. A higher proportion of “good” embryos also seemed to be concentrated where the number of oocytes retrieved was low. Additionally, with a *p* value approaching significance, 0.1025, and a negative parameter estimate, − 0.000929, in the linear regression model using the number of oocytes retrieved as dependent variable, such additional evidence may suggest that higher number of oocytes retrieved may negatively impact the rate of euploid embryos.

Studies have shown evidence suggesting that mild ovarian stimulation protocols may be associated with better-quality embryos compared to conventional protocols [8]. Higher doses of follicle-stimulating hormone (FSH) have been found to be associated with meiotic division errors in IVF embryos [9]. Shorter-duration stimulation and lower total gonadotropin dose have also been shown to produce better-quality embryos than longer-duration stimulation protocols [10].

An issue arises when looking at stimulation protocols for certain confounding conditions such as PCOS and obesity. When optimizing and standardizing gonadotropin dosing based on age and ovarian reserve markers, those with PCOS, particularly with high AMH, do not seem to garner the same results [11]. Furthermore, obesity has been shown to result in a smaller proportion of good-quality embryos. However, this same population also requires larger amounts of gonadotropins to achieve similar IVF success rates [7]. It goes to question whether lesser stimulation in such populations would in fact lead to more euploid embryos given that these specific cohorts may potentially produce lesser quality oocytes at baseline.

Nevertheless, recognizing a potentially negative impact on embryo euploid rate with higher oocyte retrieval numbers could potentially shift the landscape surrounding COS to one centered on obtaining the “appropriate” number of oocytes rather than a maximum number of oocytes. This could ultimately optimize euploidy rates, and thus the chance at a live birth, while decreasing those potential risks associated with aggressive COS protocols.

A particular limitation of this study is the lack of analysis pertaining to differing stimulation protocol characteristics, such as medications and subsequent dosages used for stimulation as well as duration of treatment. Further analysis into these parameters could shed light on whether such protocol characteristics share any association with rate of euploid embryos.

## Conclusion

Even though the number of oocytes retrieved was not found to have a statistically significant effect on the euploidy rate of embryos, there is some evidence to suggest that the higher number of oocytes retrieved may negatively impact the rate of euploid embryos. Additional research on the effect of high egg yields on embryo euploidy rates would be beneficial to further expand on these findings.

## Data Availability

The datasets used and analyzed during the current study are available from the corresponding author on reasonable request.
